# Influence of Hypothermic Machine Perfusion on Markers of Oxidative Stress and Early Tubular Injury in Rat Donor Kidneys Before Transplantation

**DOI:** 10.3390/kidneydial6020023

**Published:** 2026-04-07

**Authors:** Caleb LeGrand, Dinesh Bhattarai, Amod Sharma, Madison K McGraw, Neriman Gokden, Lee Ann MacMillan-Crow, Nirmala Parajuli

**Affiliations:** 1 Department of Pharmacology and Toxicology, University of Arkansas for Medical Sciences, Little Rock, AR 72205, USA; 2 Department of Pathology, University of Arkansas for Medical Sciences, Little Rock, AR 72205, USA

**Keywords:** static cold storage, hypothermic machine perfusion, mitochondrial respiration, kidney injury

## Abstract

**Background:**

Hypothermic machine perfusion (HMP) has been associated with reduced delayed graft function compared with static cold storage (SCS). However, the molecular mechanisms underlying these differences during cold preservation remain incompletely understood. This study compared cold-storage-related biochemical and histological changes in kidneys preserved by HMP versus SCS using a Lewis rat model prior to transplantation.

**Methods:**

Following isolation, rat kidneys were flushed with cold saline (4 °C). Left kidneys were preserved by HMP at constant flow using Belzer’s machine perfusion solution (MPS) at 4 °C, while right kidneys were stored using SCS in University of Wisconsin solution at 4 °C. After four hours of preservation, kidneys were processed for biochemical and histological analysis. Fresh biopsies were evaluated for mitochondrial complex respiration. Western blotting was performed to assess expression of NDUFS3, a complex I subunit. Histological staining for nitrotyrosine and kidney injury markers was compared across groups.

**Results:**

Mitochondrial complex respiration did not differ significantly between the SCS and HMP groups. Western blot analysis demonstrated significantly increased NDUFS3 expression in HMP-preserved kidneys compared with SCS and control kidneys. Histological evaluation revealed elevated tubular staining of nitrotyrosine and kidney injury markers in SCS kidneys relative to controls, whereas HMP preservation markedly attenuated these increases.

**Conclusions:**

HMP mitigates cold-storage-induced oxidative stress and reduces expression of kidney injury markers after four hours of preservation. These molecular findings suggest a protective effect of HMP during cold preservation. Future studies with longer preservation times and transplantation models are needed to determine whether these improvements translate into enhanced post-transplant kidney function.

## 1. Introduction

Transplantation remains the preferred treatment modality for patients with end-stage kidney disease (ESKD). Kidneys from living donors exhibit superior graft function and fewer compliations after transplantation compared with those procured from deceased donors. Nevertheless, deceased donor kidneys constitute nearly 70% of clinical transplants and must undergo cold storage (CS) techniques before transplantation to prolong viability during transportation to the recipient. Clinical evidence indicates that prolonged CS correlates with poor transplant outcomes in patients with ESKD [1]. Despite a growing demand for kidney transplantation in the United States, organ donation rates have remained relatively constant. As a result, optimizing the preservation and utilization of deceased donor kidneys has become increasingly critical. Alarmingly, up to 25% of procured kidneys are discarded annually, with CS-related injury contributing to these losses [1,2]. These challenges highlight the need to improve current CS practices.

Pre-clinical studies demonstrate that cold ischemic injury disrupts the mitochondrial structure and function [3]. Mitochondria generate ATP through the electron transport chain, which is composed of five respiratory complexes (complexes I–V) [4,5]. Electrons originating from complex I (NADH:ubiquinone oxidoreductase) or complex II (succinate dehydrogenase) are transferred to coenzyme Q, then to complex III (cytochrome c reductase), and ultimately to complex IV (cytochrome c oxidase), which reduces oxygen. Proton translocation during this process establishes the electrochemical gradient used by complex V (ATP synthase) to produce ATP. During the respiration process (oxidative phosphorylation), some electrons are leaked and form superoxide radicals, which react with nitric oxide to form peroxynitrite [6–8]. Peroxynitrite is a short-lived, highly reactive molecule that reacts with various biomolecules such as proteins, lipids, and DNA and causes oxidative damage, leading to alterations in their structure and function, contributing to cell damage [9,10]. We previously reported that 18 h of static cold storage (SCS) decreases the mitochondrial complex I and complex II respiration in rat kidneys and generates reactive oxygen species (ROS) [9,11]. Rat transplant models further show that 4 or 18 h of SCS decreases mitochondrial respiratory complex function, depletes ATP, and exacerbates tissue injury in kidney transplants [5], suggesting that SCS-induced mitochondrial dysfunction significantly contributes to poor transplant outcomes.

The transplant community has long sought improved CS techniques. Hypothermic machine perfusion (HMP) has gained prominence as an alternative to SCS for preserving deceased donor kidneys. Multiple clinical studies indicated that HMP reduces delayed graft function by over 30% compared with SCS [5]. However, the mechanistic basis for the superiority of HMP remains unclear, particularly regarding the preservation of mitochondrial function. In this study, we employed a rat kidney model using short-term CS (4 h) to compare HMP and SCS in relation to mitochondrial injury, oxidative stress, and injury markers prior to transplantation.

## 2. Materials and Methods

### 2.1 Animals

Lewis rats (8 weeks) weighing 200–250 g were obtained from Charles River Laboratories. All animal experiments were performed according to the animal use protocol approved by the University of Arkansas for Medical Sciences (UAMS) Institutional Animal Care and Use Committee and in compliance with institutional and NIH guidelines.

### 2.2 Rat Kidney Isolation and Study Design

Lewis rats (n = 8) were used, with four serving as untreated controls (2 male, 2 female) and four as cold storage donors (2 male and 2 female). Rats were anesthetized with isoflurane (5% induction and 2% maintenance) using an ISOTEC vaporizer. After the induction, the rats were removed from the induction chamber and placed on a surgical platform in the supine position. The nose was inserted inside the nose cone supplied with 2% isoflurane vapor. Body temperature was maintained at 37 °C using a heating pad. Toe pinch was performed to confirm deep surgical anesthesia. Hairs were removed, and the surgical area was prepared using 3 times alternate swabbing with betadine and 70% ethanol. A midline laparotomy was performed to expose the abdominal organs.

All kidney flushing and isolation were performed under anesthesia. For the control condition, both kidneys from each control rat were flushed with saline containing 1 U/mL heparin at room temperature, then isolated and processed as untreated control kidneys. For cold storage experiments, both kidneys from each donor rat were flushed with cold heparinized saline (1 U/mL, 4 °C).

- Static cold storage (SCS): The right kidneys (n = 4) were then flushed with and stored in University of Wisconsin (UW) solution at 4 °C for 4 h.
- Hypothermic machine perfusion (HMP): The left kidneys (n = 4) were isolated along with ~2 cm segments of the aorta and vena cava. The vessels were ligated distally with 6–0 silk sutures and canulated proximally, after which the kidneys were placed into the hypothermic machine perfusion apparatus (Harvard Apparatus, Holliston, MA, USA). Kidneys were perfused through the canulated aorta with a constant flow rate of 0.3 mL/min, and mean arterial pressure was maintained at 18.4 ± 4.9 mmHg. A total of 200 mL of recirculating Belzer MPS^®^ (Duluth, GA, USA) solution was used at 4 °C for 4 h.

Predefined organ inclusion and exclusion criteria were applied to ensure experimental consistency and technical reliability. Kidneys from animals presenting with double renal arteries or veins were excluded prior to experimentation, as successful cannulation for hypothermic machine perfusion (HMP) requires a single renal artery. Organs were also excluded if adequate flushing could not be achieved due to technical issues (e.g., incomplete removal of blood) or if significant intraoperative bleeding occurred during organ retrieval, which could compromise organ quality or ischemia time. Preparations meeting any exclusion criteria were omitted from subsequent procedures and analyses.

Kidneys from both experimental groups (SCS and HMP) were collected after 4 h and immediately processed. Fresh biopsies were used for mitochondrial function assays; tissues were fixed in formalin for histology, or flash-frozen and stored at −80 °C for subsequent biochemical analyses. Control kidneys were processed immediately after procurement. Rats were euthanized by exsanguination, and death was confirmed by removal of the heart.

### 2.3 High Resolution Respirometry

A thin cross-section of kidney at renal hilum was obtained, and two biopsies, each representing cortex and medulla, were prepared and processed for analysis of electron transport chain function using High Resolution Respirometry (HRR) (Oroboros instruments—Oxygraph-2k, Innsbruck, Austria) and the substrate–inhibitor–titration protocol (SIT) as described previously [12,13]. Briefly, saponin (100 ug/mL)-permeabilized biopsies (5–10 mg, representing cortex and medulla) of the kidneys were considered for mitochondrial complex respiration studies. Mitochondrial respiration was initiated by adding malate (2 mM) and glutamate (10 mM), and maximum active respiration was achieved by adding ADP (2.5 mM). Rotenone (0.1 mM) was then added to completely inhibit complex I respiration. To measure complex II and III respiration, succinate (10 mM) was added, followed by malonate (2 mM) to inhibit complex II respiration, and antimycin A (10 μM) to inhibit complex III respiration. To measure complex IV respiration, Tetramethyl-p-phenylenediamine (TMPD 5 μM; ascorbate-stabilized) was added followed by inhibition with 250 mM azide. Finally, data analysis was done using DATLAB 5.0 software (Oroboros, Innsbruck, Austria), and tissue respiration was shown as oxygen flux (pmol/mg/s).

### 2.4 Histology and Immunohistochemistry

Paraffin sections from untreated control, SCS (4 h), and HMP (4 h) kidneys were assessed for glomerular/tubular injury using periodic acid–Schiff (PAS) stain as described previously [14]. Histological sections were evaluated in a blinded fashion by a renal pathologist at UAMS. All parameters culminating in a tubular injury score were on a scale of 0–3, as described previously [5]. Similarly, KIM-1, NGAL, and nitrotyrosine (see Table 1 for antibody information) immunohistochemistry on renal sections were performed as described previously [15].

### 2.5 SDS-PAGE and Immunoblotting

For SDS-PAGE, renal extracts from whole-kidney homogenates were prepared with RIPA lysis buffer (Pierce, Mumbai, MA, USA). For native PAGE, renal extracts from whole-kidney homogenates were prepared with 0.9% digitonin lysis buffer as described before. Renal extracts (20–30 μg) were resolved with a Bis-Tris (4–12%) gel under denaturing (SDS-PAGE) and then transferred to a PVDF membrane. After transfer, the membrane was incubated with 1X Red Alert Western Blot Stain (Millipore) for 10 min at room temperature (to visualize protein bands), followed by blocking with blocking solution (Intercept^®^ [PBS] Protein-Free Blocking Buffer) (LICOR, Biosciences, Lincoln, NB, USA). Western blot analysis was performed with primary antibodies (Table 1); β-actin was used as a loading control. Probed membranes were washed, incubated with infrared dye-conjugated secondary goat anti-mouse or goat anti-rabbit antibodies (Table 1), and the immunoreactive bands were visualized using the Odyssey Infrared Imager and Image Studio (iS) software version 4.0 (LICOR). For all Western blot analyses, a densitometry ratio of target protein to the corresponding loading control (β-actin) was considered for statistical evaluation.

### 2.6 Statistical Analysis

To determine the necessary sample size to have a high probability of detecting a true effect, we performed a priori sample size calculation based on our studies on the kidney injury (KIM1) score between cold storage vs control conditions or SCS vs HMP conditions. Using the mean ± standard deviation, we first calculated the sample effect size (Cohen’s d), followed by calculating the sample size based on that effect. Accordingly, we got a minimum sample size of 2 and a maximum sample size of 4 for a two-tailed *t*-test for both comparisons.

A sex-balanced design was employed in all experimental groups, with equal numbers of male and female animals included to minimize potential sex-related bias. The study was not specifically powered to detect sex-dependent differences; therefore, no separate statistical analysis was performed to evaluate sex as an independent biological variable. The inclusion of balanced sexes was intended to enhance generalizability while avoiding overinterpretation of sex-specific effects.

Data (n = 4 per group) were evaluated using GraphPad Prism Version 10 and are presented as the mean ± standard error of the mean (SEM). The Shapiro–Wilk test was used to assess normality. For normally distributed data, one-way ANOVA followed by Tukey’s post hoc test was used for multiple group comparisons. For skewed data, the Mann–Whitney U test was applied to compare two groups at a 95% confidence level. Additionally, paired comparisons between SCS and HMP kidneys (from the same rat) were performed using a two-tailed paired t-test for normally distributed data, and the Wilcoxon matched-pairs signed-rank test for non-normal data. Differences were considered statistically significant at *p* < 0.05.

## 3. Results

### 3.1 Mitochondrial Respiratory Complex III Respiration Decreases After SCS or HMP

**Figure 1.**
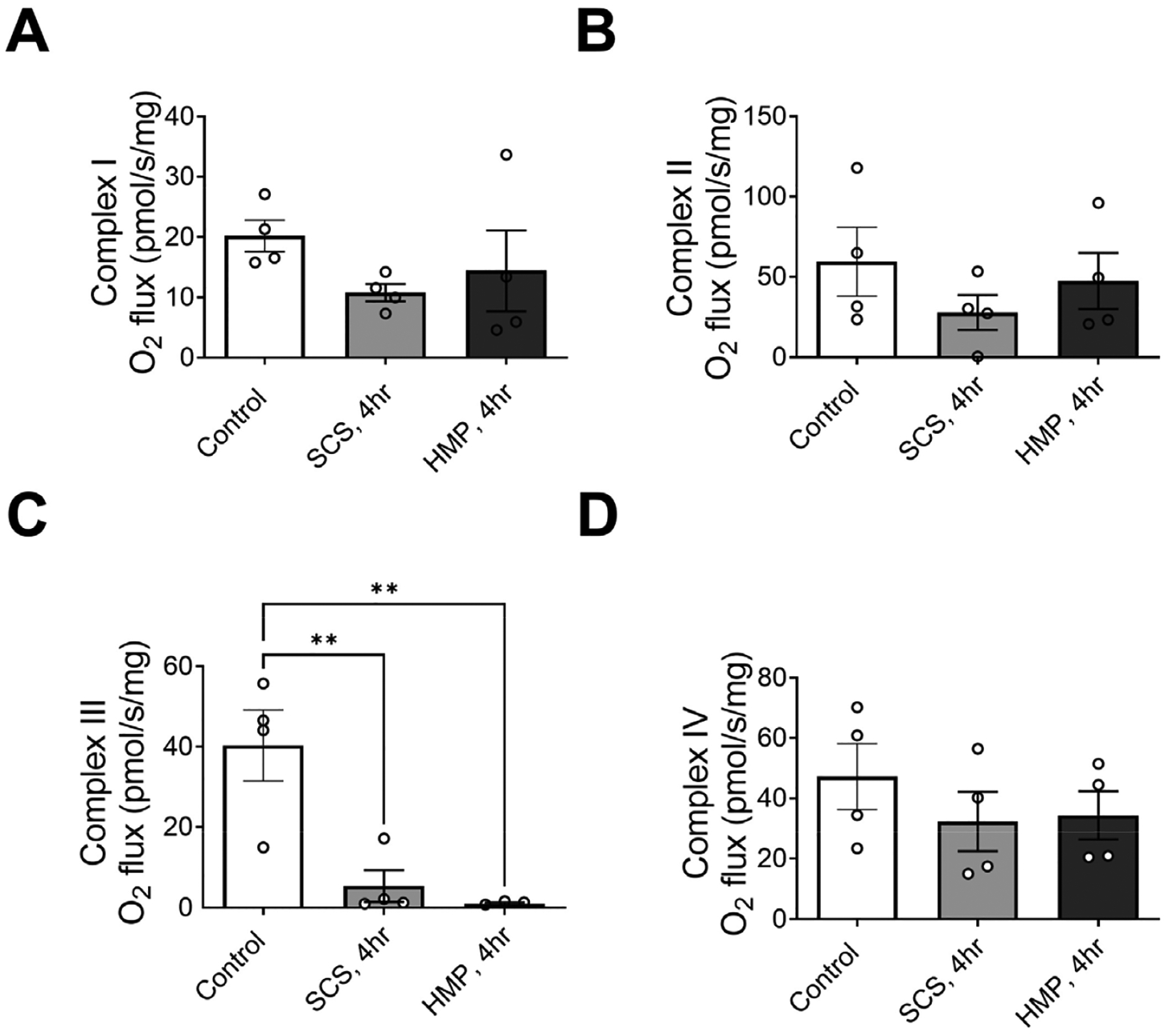
Effects of cold storage methods (static cold storage and hypothermic machine perfusion) on mitochondrial complex respiration. Isolated rat kidneys were preserved for 4 h at 4 °C using static cold storage (SCS) or hypothermic machine perfusion (HMP) methods. Untreated control kidneys were used as controls. (**A**–**D**) Mitochondrial respiratory complex (I-IV) activities were measured in rat kidney biopsies (~8 mg) using high-resolution respirometry and substrate inhibition titration protocol. Bar graphs show complex I (**A**), complex II (**B**), complex III (**C**), and complex IV (**D**) activities. Data are presented as mean ± SEM (n = 4 per group), and statistical evaluation was performed using one-way ANOVA (GraphPad Prism, Version 10). ** *p* < 0.01.

Rat kidneys were assigned to either SCS or HMP. For SCS, rat kidneys were stored in cold University of Wisconsin solution at 4 °C for 4 h. HMP was performed using machine perfusion solution (MPS) at 4 °C with a flow rate of 0.3 mL/min and an average arterial pressure of 18.4 ± 4.9 mmHg. We previously showed that 4 h of SCS prior to transplantation induces mitochondrial dysfunction and renal injury in rat kidneys. Here, we assessed mitochondrial respiratory complex activities after 4 h of SCS or HMP without transplantation. Fresh renal biopsies were analyzed using the Oxygraph 2K (Oroboros Instruments, Innsbruck, Austria) and the substrate–inhibitor–titration (SIT) protocol. Both SCS and HMP significantly decreased complex III respiration (Figure 1C), while activities of complexes I, II, and IV remained unchanged across groups (Figure 1A,B,D).

**Figure 2.**
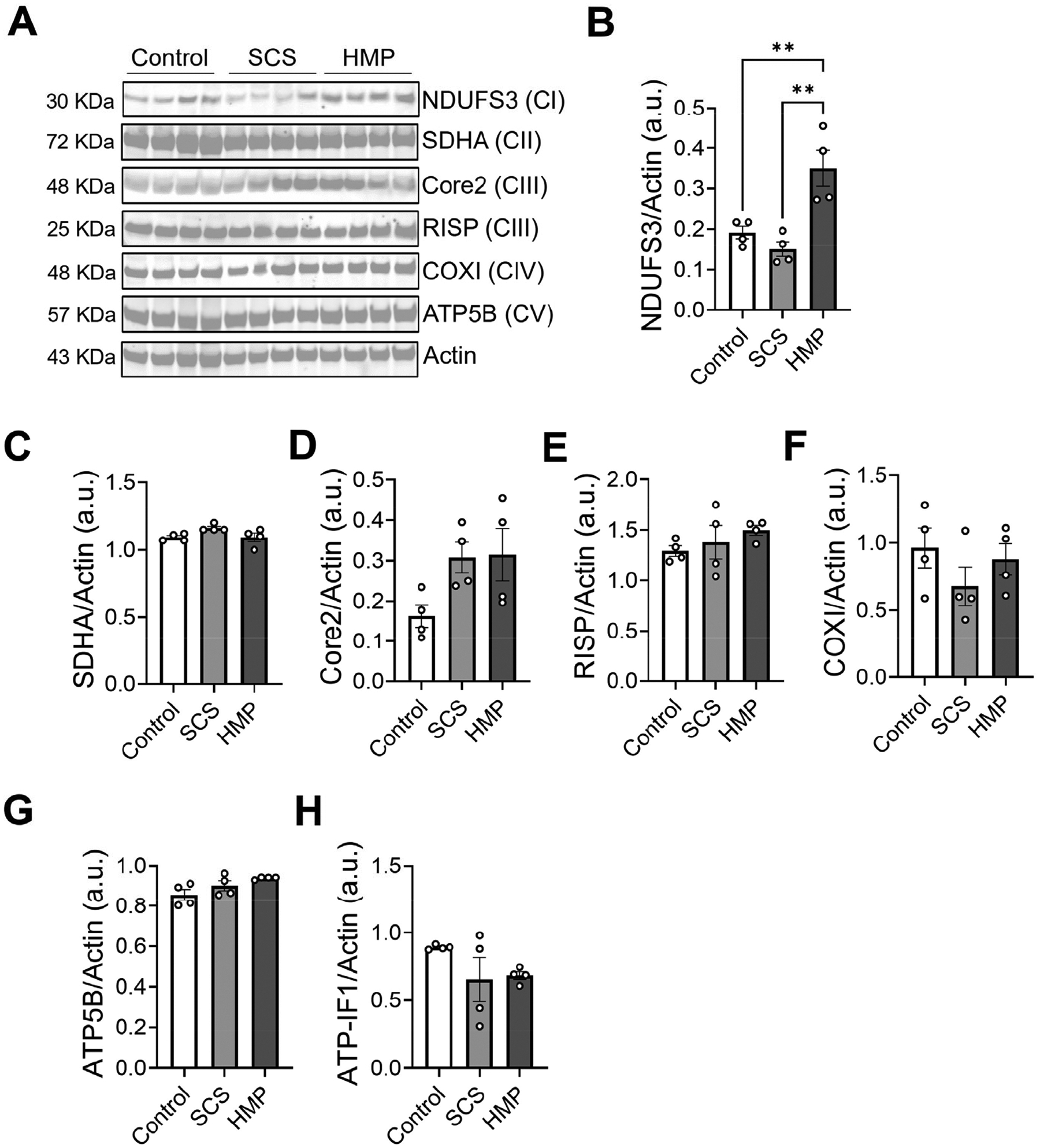
Effects of cold storage methods (static cold storage and hypothermic machine perfusion) on mitochondrial protein levels. Isolated rat kidneys were preserved for 4 h at 4 °C using static cold storage (SCS) or hypothermic machine perfusion (HMP) methods. Untreated control kidneys were used as control. (**A**–**H**) RIPA lysates were prepared from rat kidney homogenates of control, SCS, and HMP groups, followed by SDS-PAGE Western blotting of respiratory complex subunits. β-actin was used as a control. (**A**) Representative Western blot images are shown. (**B**–**H**) Bar graph showing densitometry ratio of indicated respiratory subunits and β-actin: NDUFS3 (**B**), SDHA (**C**), Core2 (**D**), RISP (**E**), COXI (**F**), ATP5B (**G**), and ATP-IF1 (**H**). Data are presented as mean ± SEM (n = 4 per group), and statistical evaluation was performed using one-way ANOVA (GraphPad Prism, Version 10). ** *p* < 0.01.

Next, we evaluated the expression of representative subunits of mitochondrial complexes I–V using Western blotting (Figure 2; Table 1). The complex I subunit NDUFS3 remained unchanged in SCS kidneys compared with controls but was significantly increased in HMP kidneys relative to both control and SCS groups (Figure 2A,B). The expression of SDHA (complex II), Core 2 and RISP (complex III), COXI (complex IV), and ATP5B (complex V) did not differ among groups (Figure 2A,C–H).

### 3.2 HMP Reduces Oxidative Stress in Donor Rat Kidneys

**Figure 3.**
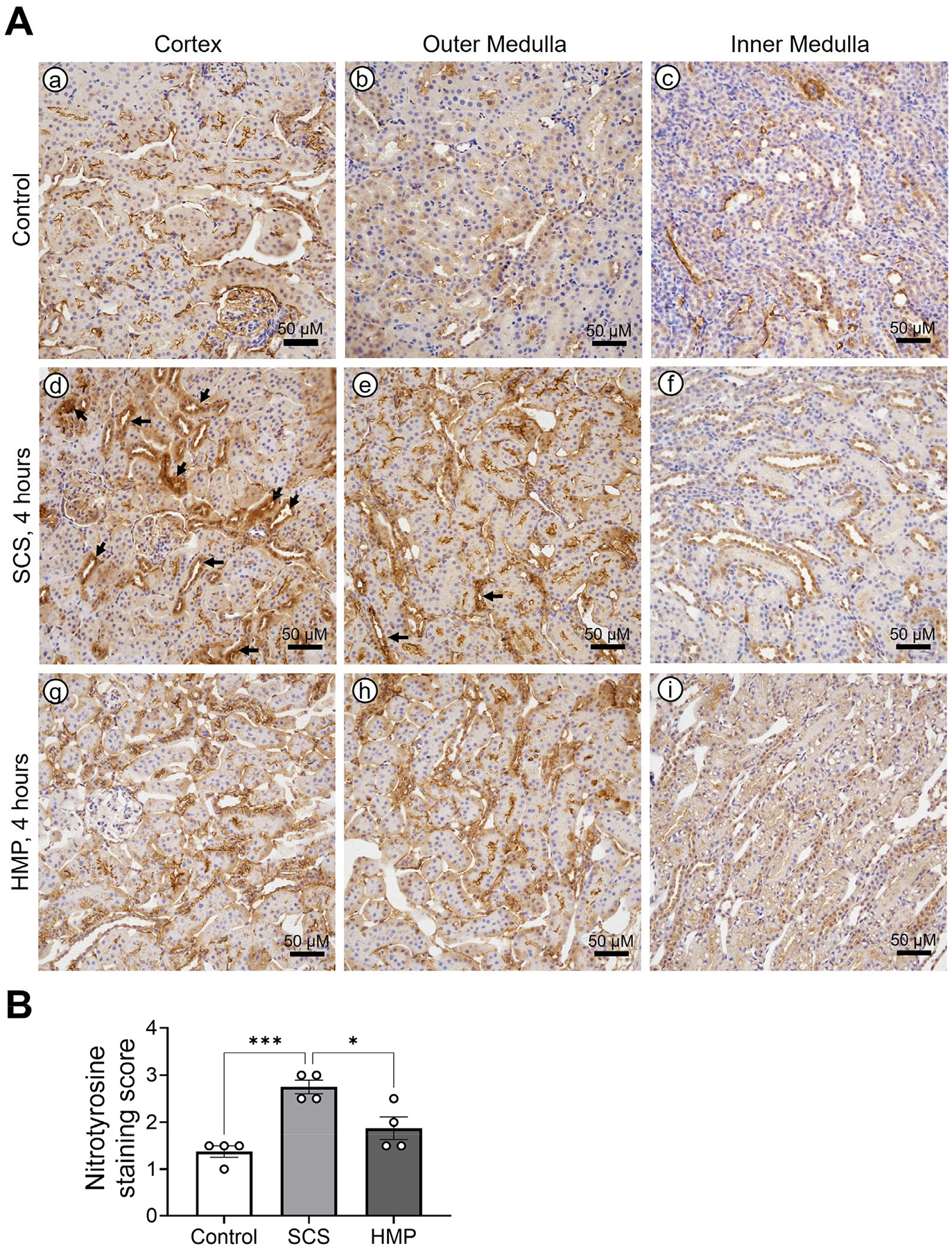
Effects of cold storage methods (static cold storage and hypothermic machine perfusion) on oxidative stress. Rat kidney sections derived from untreated control, static cold storage (SCS), and hypothermic machine perfusion (HMP) groups were employed for nitrotyrosine (a marker of reactive oxygen species) immunohistochemistry. (**A**) Representative immunohistochemical images of nitrotyrosine staining of control (**a**–**c**), 4 h SCS (**d**–**f**), and hypothermic machine perfusion (**g**–**i**) kidneys. Scale bar = 100 μm. (**B**) Bar graph showing semi-quantitative analysis of nitrotyrosine levels across experimental groups. Data are presented as mean ± SEM (n = 4 per group). Statistical significance was determined by one-way ANOVA (GraphPad Prism, Version 10). * *p* < 0.05; *** *p* < 0.001.

Because SCS increases mitochondrial superoxide levels in donor kidneys, we examined oxidative stress by assessing nitrotyrosine staining. Control kidneys showed basal nitrotyrosine levels (Figure 3A(a–c)). After 4 h of SCS, nitrotyrosine staining markedly increased (Figure 3A(d–f)), whereas HMP kidneys showed only a modest increase compared with controls and significantly less staining than SCS kidneys (Figure 3A(g–i)). Semiquantitative scoring confirmed significantly elevated nitrotyrosine in SCS kidneys verus controls and a significant reduction in HMP kidneys versus SCS (Figure 3B), indicating that HMP mitigates CS-induced oxidative stress.

**Figure 4.**
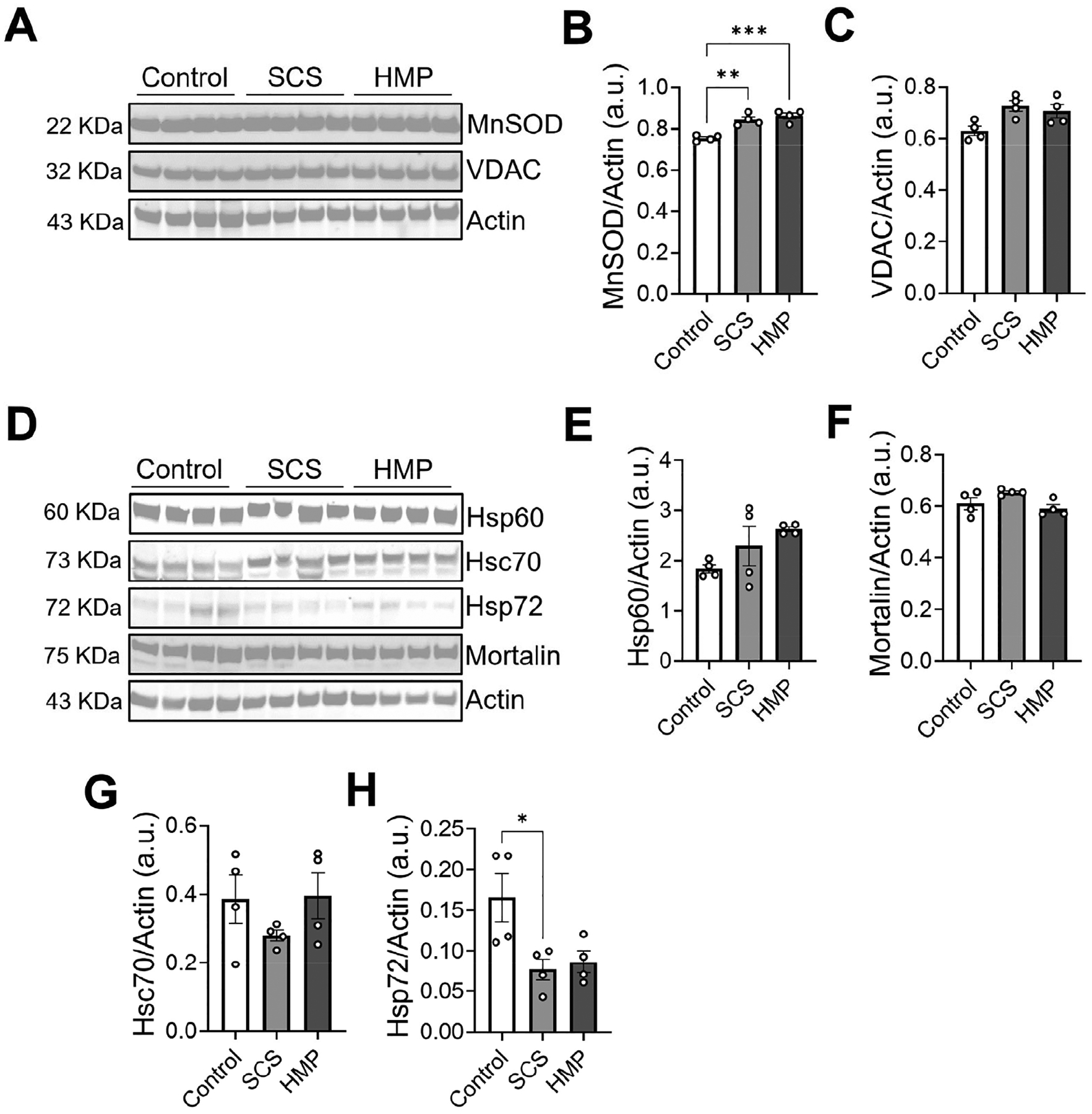
Effects of cold storage methods (static cold storage and hypothermic machine perfusion) on antioxidant enzymes and heat shock chaperones. (**A**–**H**) RIPA lysates were prepared from rat kidney homogenates of control, static cold storage (SCS), and hypothermic machine perfusion (HMP) groups, followed by SDS-PAGE Western blotting. (**A**) Representative Western blot images of MnSOD and VDAC are shown. β-actin was used as a control. (**B**,**C**) Bar graph showing densitometry analysis of MnSOD (B) or VDAC (**C**) normalized to β-actin. (**D**) Representative Western blot images of heat shock chaperones (Hsp60, Hsc70, Hsp72, and mortalin) and β-actin (loading control) are shown. (**E**–**H**) Bar graphs showing densitometry analyses of Hsp60 (**E**), mortalin (**F**), Hsc70 (**G**), and Hsp72 (**H**) normalized to β-actin. Data are presented as mean ± SEM (n = 4 per group), and statistical evaluation was performed using one-way ANOVA (GraphPad Prism, Version 10). * *p* < 0.05; ** *p* < 0.01; *** *p* < 0.001.

Because HMP reduced nitrotyrosine levels in kidneys, we sought to assess manganese superoxide dismutase (MnSOD), a superoxide scavenger located in mitochondria. Western blot analysis showed a significant change in MnSOD levels in both SCS and HMP groups relative to controls (Figure 4A,B). In contrast, VDAC, an outer mitochondrial membrane-localized protein responsible for transporting ions/metabolites between the cytosol and the inner mitochondrial membrane, remained unchanged among all groups. (Figure 4A,C).

Stress, including ROS, induces oxidative damage to proteins and thereby influences the function of a diverse array of cellular processes. Molecular chaperones are a class of proteins that identify damaged proteins, inhibit protein aggregation by binding to the damaged proteins, correct their folding, or triage them for proteasomal or autophagolysosomal degradation [14]. To evaluate whether 4 h of SCS or HMP affects the dysregulation of molecular chaperones, we assessed heat shock proteins. SCS or HMP did not alter the levels of mitochondrial heat shock proteins, namely Hsp60 (Figure 4D,E) and mortalin (Figure 4D,F), or cytosolic heat shock protein Hsc70 in donor rat kidneys (Figure 4D,G). Unexpectedly, a significant decrease in Hsp72 protein was observed in the SCS kidneys compared to the control, and a modest decrease in this protein was observed after HMP (Figure 4D,H).

### 3.3 Renal Morphological Changes Are Not Significantly Different Between SCS and HMP

**Figure 5.**
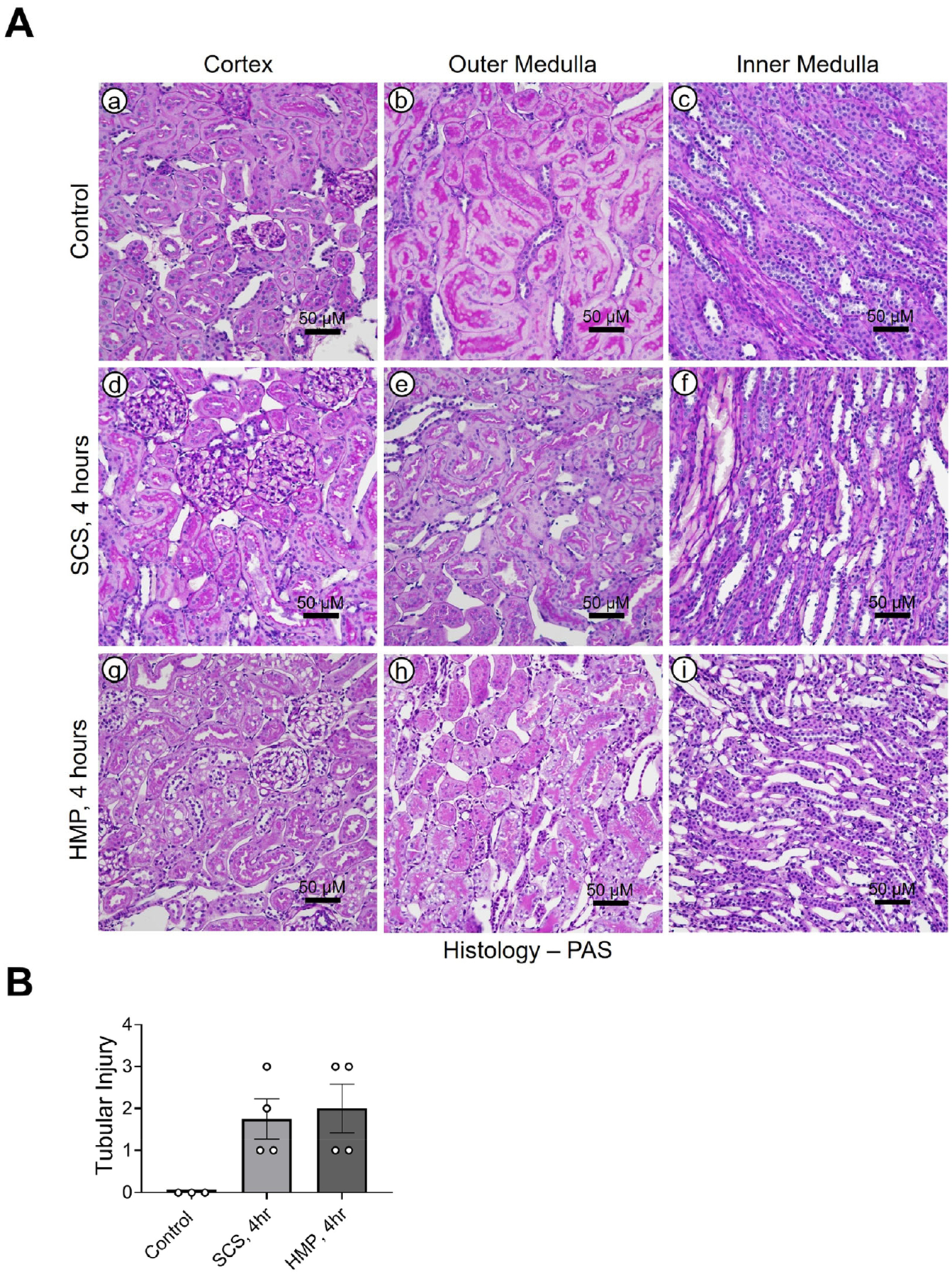
Effects of cold storage methods (static cold storage and hypothermic machine perfusion) on kidney injury. Formalin-fixed paraffin-embedded kidney sections (4–5 μm) derived from control, static cold storage (SCS), and hypothermic machine perfusion (HMP) groups were considered for histological evaluations by periodic acid–Schiff (PAS) staining. (**A**) Representative micrographs (400×) of PAS-stained kidney sections from control (**a**–**c**), SCS (**d**–**f**), and HMP (**g**–**i**). Scale bar = 100 μm. (**B**) Bar graphs showing pathological evaluation of tubular injury score performed by a blinded renal pathologist. Data are expressed as mean ± SEM (n = 3–4 per group). Statistical significance was determined by one-way ANOVA.

**Figure 6.**
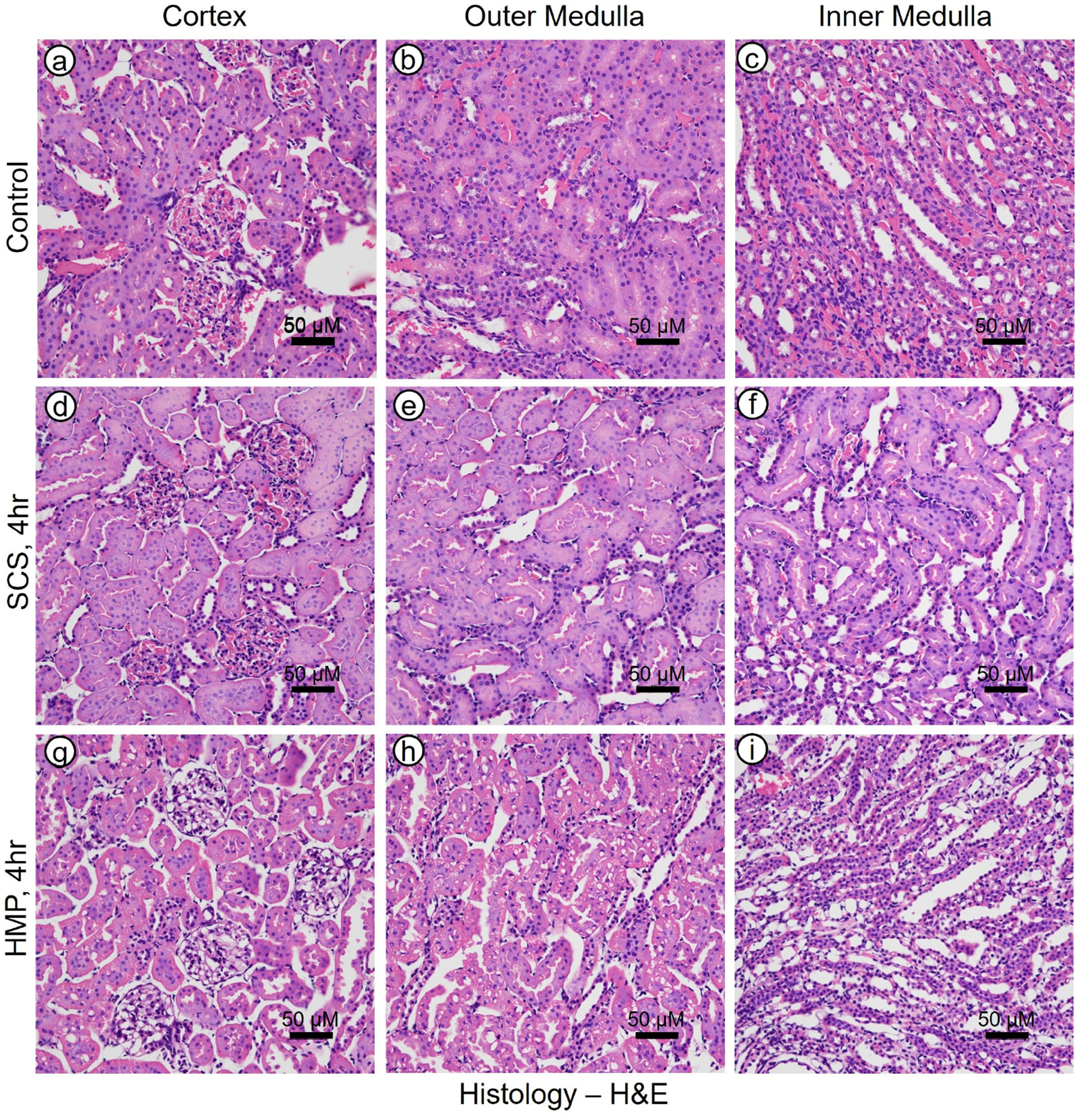
Effects of cold storage methods (static cold storage and hypothermic machine perfusion) on kidney morphology. Formalin-fixed paraffin-embedded kidney sections (4–5 μm) derived from control, static cold storage (SCS), and hypothermic machine perfusion (HMP) groups were considered for histological evaluations by hematoxylin and eosin (H&E) staining. Representative micrographs (400×, n = 3–4 per group) showing H&E-stained kidney sections from control (**a**–**c**), SCS (**d**–**f**), and HMP (**g**–**i**). Scale bar = 100 μm. No changes in renal morphology were noted by a blinded renal pathologist.

To compare the levels of CS-mediated injury, kidney sections derived from formalin-fixed paraffin-embedded kidney blocks after SCS or HMP were employed for histological evaluations using PAS and H&E staining. Morphological changes between SCS, HMP, and control groups were identified by a blinded renal pathologist. For both HMP and SCS, broad ischemic changes were noted in the PAS-stained kidney sections, including loss of epithelial brush border in the proximal tubules, but the scoring did not show a significant difference between the HMP and SCS groups (Figure 5A,B). Similarly, H&E stain did not reveal any signs of morphological changes or inflammation in the kidneys after SCS or HMP compared to the control group (Figure 6).

### 3.4 Early Tissue Injury Markers Are Increased After SCS; HMP Blunts Tissue Injury During CS

**Figure 7.**
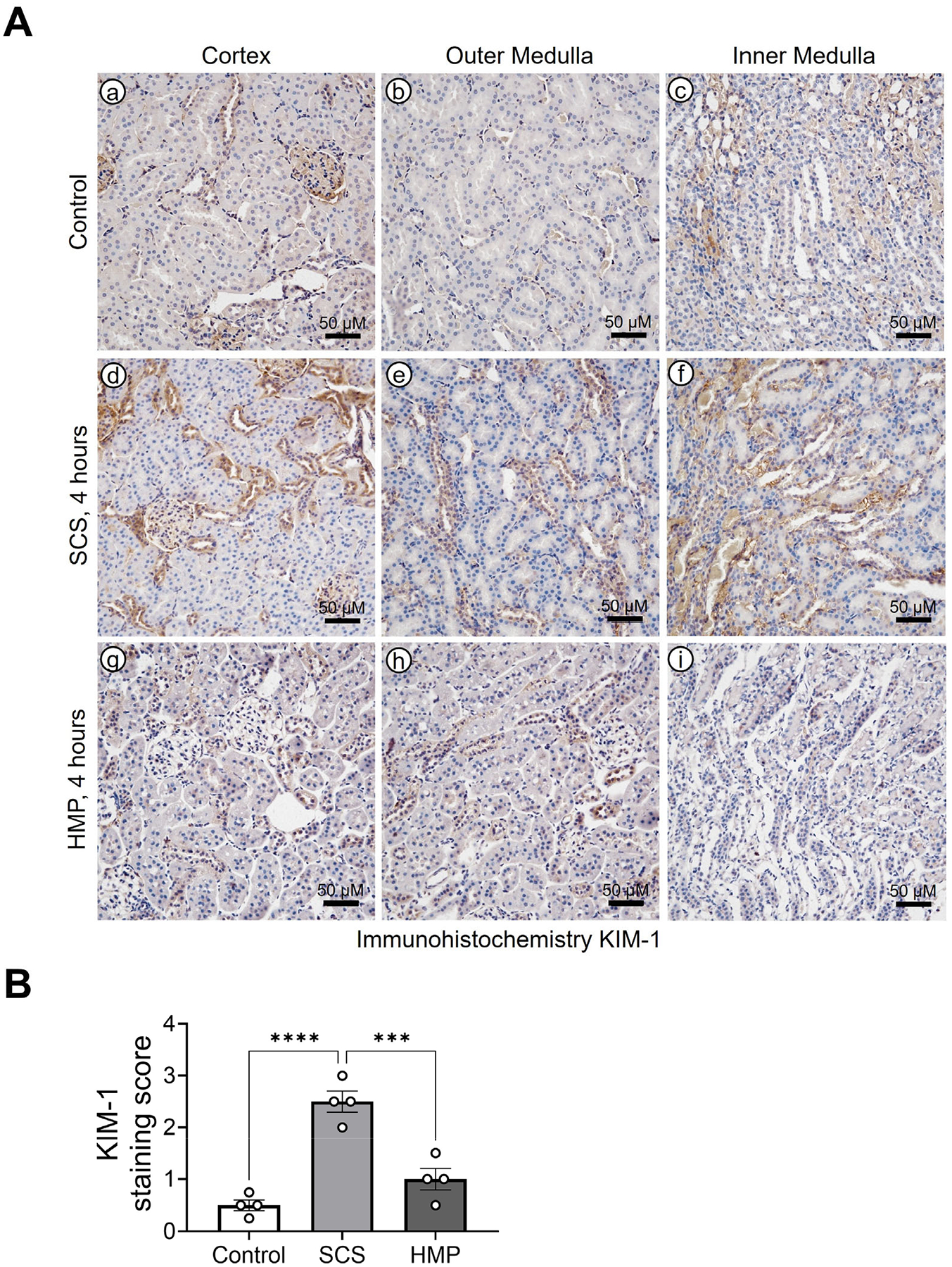

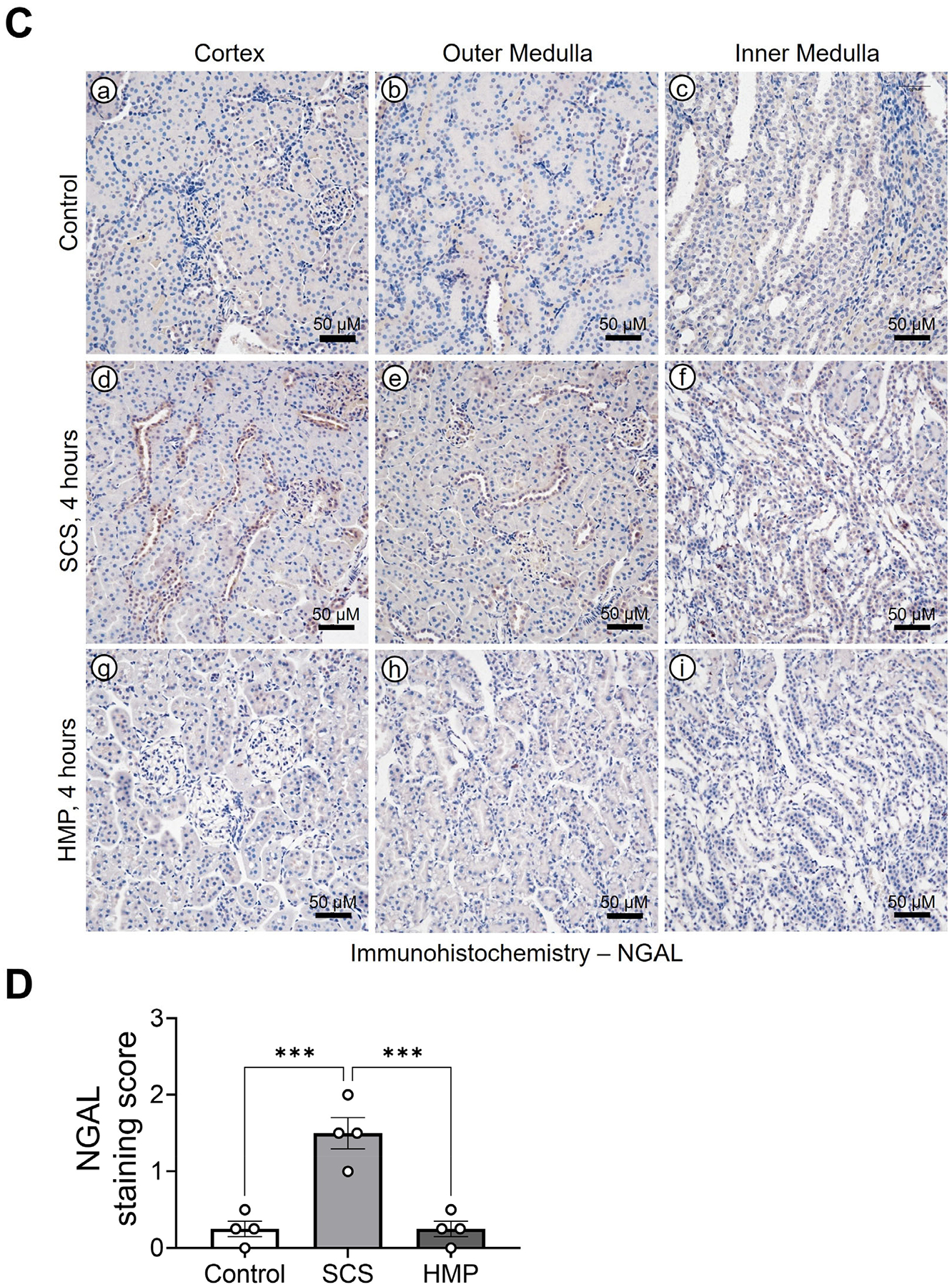
Effects of cold storage methods (static cold storage and hypothermic machine perfusion) on kidney injury markers. Formalin-fixed paraffin-embedded kidney sections (4–5 μm) derived from control, static cold storage (SCS), and hypothermic machine perfusion (HMP) groups were considered for kidney injury molecule-1 (KIM-1) and neutrophil gelatinase-associated lipocalin (NGAL) immunohistochemistry, followed by imaging (400×) and semi-quantitative scoring. (**A**) Representative micrographs showing KIM-1-positive renal tubules (brown stain) from control (**a**–**c**), SCS (**d**–**f**), and HMP (**g**–**i**) kidney sections. Scale bar = 100 μm. (**B**) Bar graphs showing semi-quantitative score of KIM-1 staining. Data are expressed as mean ± SEM (n = 4 per group). Statistical significance was determined by one-way ANOVA (GraphPad Prism, Version 10). *** *p* < 0.001; **** *p* < 0.0001. (**C**) Representative micrographs showing NGAL-positive renal tubules (brown stain) from control (**a**–**c**), SCS (**d**–**f**), and HMP (**g**–**i**) kidney sections. Scale bar = 100 μm. (**D**) Bar graphs showing semi-quantitative score of NGAL staining. Data are expressed as mean ± SEM (n = 4 per group). Statistical significance was determined by one-way ANOVA (GraphPad Prism, Version 10). *** *p* < 0.001.

Because routine histochemical evaluation could not distinguish injury severity between groups, we assessed early injury markers KIM1 and NGAL using immunohistochemistry. Control kidneys displayed minimal staining for KIM 1 (Figure 7A(a–c)) and NGAL (Figure 7C(a–c)). As expected, SCS kidneys exhibited strong tubular staining for both KIM1 or NGAL (Figure 7A,C). In contrast, HMP kidneys showed markedly reduced staining compared with SCS. Semiquantitative scoring confirmed significant increases in KIM1 and NGAL after SCS, while HMP significantly lowered both markers (Figure 7B,D), demonstrating that HMP mitigates early CS-induced kidney injury [16].

## 4. Discussion

SCS is a simple CS technique that is widely used to preserve deceased donor kidneys, yet prolonged CS negatively impacts graft outcomes [17,18]. Clinical studies show that HMP improves transplantation results and reduces delayed graft function (DGF) [1,2]. However, the molecular differences underlying these preservation techniques are not well defined. Buidling upon our prior findings that SCS exacerbates mitochondrial dysfunction after transplantation [12,13], we compared SCS and HMP under controlled pre-transplant (4 h) ex vivo conditions.

We observed that both SCS and HMP reduced mitochondrial complex III respiration, while complexes I, II, and IV were preserved. This finding contrasts with prior studies showing more widespread mitochondrial suppression after longer SCS durations or after reperfusion [5,14]. Collectively, these findings further suggest that oxidative stress and early tissue injury are the SCS-derived factors that might promote delayed graft function and perpetuate transplant injury. Future studies are needed to evaluate the consequences of SCS- and HMP-derived molecular changes on graft outcome following transplantation using the established rat models.

The mitochondrial electron transport chain is the major intracellular site of superoxide production under normal or pathological conditions in eukaryotic cells [5]. Complex I and complex III are major sites of superoxide formation in mitochondria [10,19]. This study showed that 4 h of both SCS and HMP decreased only complex III respiration in rat kidneys, which was an unexpected finding. Previous experimental studies have demonstrated that SCS significantly impairs mitochondrial function, particularly affecting complex respiration and ATP production in renal grafts. In a rat kidney transplantation model, 18 h of SCS compromised complex I and II respiration, while SCS combined with transplantation reduced complex I, II, and III respiration 1 day post-transplant [20]. Similarly, 4 h of SCS followed by transplantation led to a marked reduction in complex I and III respiration, along with a significant drop in ATP levels 1 day post-transplant when compared to sham or autotransplant controls [21]. In vivo cold ischemia–reperfusion studies in rats also reported MnSOD inactivation and global suppression of complexes I-IV in kidneys, highlighting the vulnerability of the oxidative phosphorylation to cold ischemia-mediated injury [5].

Interestingly, in a porcine kidney model, complex II+III respiration increased after 48 h of SCS compared to 24 h, although the study lacked baseline control measurements. This suggests a dynamic response of mitochondrial complexes during prolonged SCS. Additionally, cold HMP has been shown to reduce oxygen consumption while increasing lipid peroxidation and hydrogen peroxide production in porcine kidneys [14]. Notably, controlled oxygenated rewarming following prolonged cold preservation improves mitochondrial coupling efficiency, spare respiratory capacity, and oxygen utilization upon reperfusion, indicating partial restoration of oxidative metabolism. Together, these findings underscore the dynamic nature of mitochondrial respiration, injury, and recovery during cold preservation and following transplantation.

A notable finding was the HMP-induced increase in NDUFS3, a core subunit of mitochondrial complex I. NDUFS3 is encoded by the *Ndufs3* gene, one of the 13 genes of the mitochondria [22], and is ubiquitously expressed across multiple organs, including the kidney. Previous studies have implicated NDUFS3 in protective mitochondrial responses, including reduced ferroptosis in renal tubular cells and increased expression in diabetic nephropathy. In human kidney proximal tubular cells, overexpression of NDUFS3 has been shown to attenuate the LPS-mediated ferroptosis and mitochondrial injury [18]. Similarly, NDFUS3 expression has been reported in the glomeruli of diabetic kidneys [23]. As a core subunit of complex I, NDUFS3 plays an essential role in assembly and stabilization of iron–sulfur cluster-containing subunits and supports conformational coupling required for efficient electron transfer [4,5]. In this context, the observed increase in NDUFS3 expression in HMP-treated kidneys was associated with reduced nitrotyrosine levels and lower expression of kidney injury markers (KIM1/NGAL), suggesting a potential role in limiting electron leak and oxidative stress. However, despite increased NDUFS3 expression, complex I respiratory activity was not restored, consistent with the fact that complex I is a large multimeric enzyme comprising approximately 45 subunits. Therefore, increased expression of a single subunit may reflect a compensatory or adaptive response rather than functional recovery. Future studies should focus on comprehensive analyses of complex I subunit expression, assembly, and structural integrity, with particular attention to the role of NDUFS3 in HMP-mediated mitochondrial protection.

KIM-1 and NGAL are widely used as sensitive early biomarkers of tubular injury, but accumulating evidence indicates that they also participate in key injury-response and repair mechanisms. KIM-1, which is minimally expressed in healthy kidneys [24], is rapidly upregulated in proximal tubular epithelial cells following ischemic or toxic injury and contributes to epithelial recovery by mediating phagocytic clearance of apoptotic debris and modulating innate immune activation [25]. Likewise, NGAL, one of the earliest and most highly induced genes after tubular injury, exerts renoprotective effects through iron sequestration and reduction in oxidative stress [26]. Transcriptomic studies in rodent models of ischemia–reperfusion injury have identified NGAL as one of the most upregulated genes in the kidney early after tubular damage [27], supporting its utility as an early injury marker. Similar to KIM-1, NGAL is believed to be reno-protective during ischemic kidney injury [28,29]. In our study, kidneys preserved by HMP exhibited attenuated KIM-1 and NGAL expression compared with other preservation modalities, suggesting a reduction in early tubular stress and a decreased need to activate compensatory protective pathways. Collectively, these findings support the concept that HMP not only mitigates tubular injury but also suppresses early injury-response signaling. Framing KIM-1 and NGAL within their dual roles, as injury markers and mediators of epithelial repair, provides a more comprehensive interpretation of their expression patterns and reinforces the mechanistic basis for the protective benefits of HMP.

Clinically, HMP consistently reduces DGF and acute tubular necrosis, and improves early graft function [13,30–33]. Our findings provide mechanistic support for these observations by demonstrating differential effects on oxidative stress, mitochondrial respiratory responses, and early injury markers. Furthermore, they underscore the potential for future studies targeting mitochondrial and oxidative pathways to maintain metabolic homeostasis and facilitate waste clearance during preservation [34], which may ultimately enhance long-term graft survival and patient outcomes.

A limitation of this study is the 4 h CS period, which is shorter than typical clinical CS times (18–24 h). However, early molecular injury occurs in this window, and HMP requires continuous monitoring, making longer perfusions challenging in experimental settings. Our study provides insight into early molecular events that may set the stage for later injury.

Expanding the use of extended criteria donors and increasing the donor pool are effective strategies to improve the kidney transplant rate, which will effectively shorten the waiting time for ESKD patients. In recent years, there has been a constant search for new organ preservation techniques to prolong the viability of kidneys derived from deceased donors. Besides altering the constituents of CS solution, normothermic and oxygenated hypothermic perfusion techniques have been experimented with using pre-clinical ex vivo or in vivo models [35–38]. Although normothermic machine perfusion (NMP) is gaining popularity in steatotic hepatic organ preservation, NMP has produced mixed results for kidney preservation under experimental conditions. A study showed that NMP increases the rates of apoptosis in kidneys compared to HMP [39]. Another study using a porcine autotransplantation model with NMP and SCS kidneys showed that NMP increased proteins related to mitochondrial metabolic pathway, including oxidative phosphorylation [40]. Oxygenated perfusion preservation in particular appears promising for supporting mitochondrial function and preserving renal architecture [41–43]. Carefully designed comparative studies are needed to determine optimal preservation strategies for clinical translation.

## 5. Conclusions

Using a rat kidney cold storage model, we demonstrate that HMP reduces oxidative stress and attenuates early tissue injury compared with SCS. These findings provide molecular evidence supporting the superiority of HMP in maintaining mitochondrial stability, reducing ROS formation, and limiting tubular injury. Future in vivo studies incorporating transplantation will be essential to determine how these early molecular differences translate into functional graft outcomes.

## Figures and Tables

**Table 1.** Antibodies used for Western blotting.

Antibody	Source	Catalog Number	Dilution
Western Blotting			
Primary Antibodies			
Anti-NDUFS3 (Complex I subunit)	Abcam (Cambridge, UK)	110246	1:1000
Anti-SDHA (Complex II subunit)	Abcam	14715	1:1000
Anti-Core II (Complex III subunit)	Abcam	14745	1:1000
Anti-RISP (Complex III subunit)	Abcam	14746	1:1000
Anti-COX I (Complex IV subunit)	Abcam	14705	1:1000
Anti-ATP5B (Complex V subunit)	Invitrogen (Carlsbad, CA, USA)	A-21351	1:1000
Anti-ATPase IF1 (Complex V subunit)	Abcam	ab110277	1:1000
Anti-MnSOD	Sigma Millipore (Burlington, MA, USA)	06–984	1:1000
Anti-VDAC1/2	Proteintech (Rosemont, IL, USA)	10866–1-AP	1:1000
Anti-Hsp60	Cell Signaling Technology (Danvers, MA, USA)	12165	1:1000
Anti-Hsc70	Invitrogen	MA3–014	1:1000
Anti-Hsp72	LSBio (Newark, CA, USA)	LS-C82983	1:1000
Anti-Mortalin	Invitrogen	MA5–44603	1:1000
Anti-Actin	Invitrogen	MA5–15739	1:1000
Secondary Antibodies			
IRDye^®^ 800CW Goat anti-Mouse IgG Secondary Antibody	LICORbio^™^ (Lincoln, NE, USA)	925–32210	1:30,000
IRDye^®^ 680RD Goat anti-Rabbit IgG Secondary Antibody	LICORbio^™^	925–68071	1:30,000
Immunohistochemistry			
Primary Antibodies			
Anti-KIM1	LSBio	A10954	1:1000
Anti-NGAL	LSBio	C407821	1:1000
Anti-Nitrotyrosine	Millipore (Burlington, MA, USA)	06–284	1:500
Secondary Antibodies			
ImmPRESS^®^ HRP Horse Anti-Mouse IgG, Rat adsorbed Polymer Detection Kit, Peroxidase	Vector (Pune, MA, USA)	MP-7422–15	Kit instruction

## Data Availability

All data associated with this study are present in the paper. Data can be shared upon reasonable request to the corresponding authors.

## Abbreviations

The following abbreviations are used in this manuscript:

HMP: Hypothermic machine perfusion
SCS: Static cold storage
CS: Cold storage
UW: University of Wisconsin
NDUFS3: NADH dehydrogenase [ubiquinone] iron–sulfur protein 3
ESKD: End-stage kidney disease
ATP: Adenosine triphosphate
ADP: Adenosine diphosphate
DNA: Deoxyribonucleic acid
ROS: Reactive oxygen species
PAS: Periodic acid–Schiff stain
KIM-1: Kidney injury molecule 1
NGAL: Neutrophil gelatinase-associated lipocalin
H&E: Hematoxylin and Eosin
MPS: Machine perfusion solution
SDS-PAGE: Sodium dodecyl sulfate polyacrylamide gel electrophoresis
SIT: Substrate inhibition titration
Hsp60: Heat shock protein 60
Hsc70: Heat shock cognate70
MnSOD: Manganese superoxide dismutase
VDAC: Voltage dependent anion channel
